# Development of CAR Exosomes Targeting FAP for the Treatment of Intrauterine Adhesion

**DOI:** 10.1002/jev2.70284

**Published:** 2026-04-30

**Authors:** Wenyan Fu, Kewen Qian, Hongru Ai, Yitan Zou, Yaping Zhou, Ruixue Mao, Jian Zhao, Changhai Lei, Shi Hu

**Affiliations:** ^1^ Department of Assisted Reproduction Shanghai Ninth People's Hospital Shanghai Jiao Tong University School of Medicine Shanghai China; ^2^ Department of Biomedical Engineering College of Basic Medical Sciences Second Military Medical University Shanghai China; ^3^ Department of Biophysics College of Basic Medical Sciences Second Military Medical University Shanghai China

**Keywords:** immunotherapy, CAR‐T, exosomes, FAP, intrauterine adhesions

## Abstract

Engineered extracellular vesicles (EVs) have emerged as promising cell‐free platforms for immunomodulation and tissue repair. In this study, we generated EVs derived from chimeric antigen receptor (CAR) T cells targeting fibroblast activation protein (FAP) and investigated their biological and therapeutic functions. These FAP‐CAR EVs effectively inhibited intrauterine fibrosis, promoted endometrial regeneration, and increased pregnancy rates in a mouse model of intrauterine adhesion. Importantly, the exosome‐based therapy did not affect embryonic development or trigger systemic inflammation, indicating high safety compared with T‐cell‐based treatment. Mechanistically, while FAP‐targeted T cells could suppress fibrosis, they also induced severe cytokine‐release toxicity, which was completely avoided in the EV‐based strategy. Together, these findings demonstrate that FAP is a critical target for fibrotic disease intervention and that CAR‐T‐derived EVs represent a safe and effective vesicle‐based therapeutic modality.

## 1 Introduction

Extracellular vesicles (EVs) have recently emerged as critical mediators of intercellular communication and as promising therapeutic carriers capable of recapitulating the biological functions of their parental cells while offering superior safety and controllability. The ability of small extracellular vesicles (sEVs) to transfer functional proteins, lipids, and nucleic acids has attracted increasing attention in immunotherapy, tissue regeneration, and fibrotic disease modulation. Building upon these advances, we previously developed engineer EVs derived from chimeric antigen receptor (CAR)—modified T cells to achieve targeted, cell‐free immunomodulation (Fu et al. 2019). Such CAR‐T‐cell‐derived EVs preserved the antigen‐recognition and cytotoxic features of CAR T cells while eliminating systemic toxicity associated with cellular therapies.

Fibrotic diseases, a hallmark of ageing, impose a considerable burden on global health. These disorders are characterized by uncontrolled and progressive accumulation of fibrotic tissue in affected organs, leading to dysfunction and eventual organ failure. One representative example is intrauterine adhesion (IUA), also known as Asherman's syndrome (AS), a gynaecological disorder characterized by fibrosis and scar formation within the uterine cavity. This pathological condition often results from trauma to the endometrial lining, commonly due to surgical interventions such as dilation and curettage (D&C), caesarean section, or intrauterine device (IUD) placement (Gargett and Healy 2011). Other contributing factors include infections, postpartum complications, and chronic endometritis. IUA can lead to menstrual irregularities, infertility, recurrent pregnancy loss, and poor reproductive outcomes, severely impacting a patient's quality of life and posing significant challenges in reproductive medicine. Given the increasing incidence of IUA, particularly in women of reproductive age, there is an urgent need to explore novel therapeutic strategies that go beyond conventional treatments.

At present, the management of IUA primarily relies on surgical adhesiolysis, typically performed via hysteroscopic resection, followed by hormonal therapy and mechanical interventions such as intrauterine balloon placement to prevent re‐adhesion (Conforti et al. 2013). Although these treatments can restore partial uterine function, they have several limitations, including high recurrence rates, insufficient endometrial regeneration, and variable success in improving fertility outcomes (Keyhanvar et al. 2021). Studies report that even after hysteroscopic adhesiolysis, the recurrence rate of IUA can reach 21.8%–41.9%, particularly in cases of severe fibrosis (Hanstede et al. 1561). Hormonal therapy, often administered postoperatively, aims to promote endometrial proliferation but lacks standardized protocols and has shown inconsistent efficacy in preventing recurrence. Given these challenges, there is an urgent need for alternative and more effective therapeutic approaches that not only remove adhesions but also address the underlying fibrotic mechanisms contributing to IUA. The pathophysiology of IUA involves an imbalance among tissue injury, inflammation, and fibrosis, leading to aberrant wound healing. Fibrosis, a key feature of IUA, results from excessive deposition of extracellular matrix (ECM) components, including collagen and fibronectin, driven by dysregulated inflammatory and immune responses (Zhao et al. 2022). Recent studies have identified several molecular pathways associated with IUA pathogenesis, including transforming growth factor‐beta (TGF‐β) signalling, fibroblast activation, and immune cell infiltration (Leung et al. 2021). These findings suggest that targeting fibrosis‐related pathways could be a promising therapeutic strategy for preventing adhesion formation and promoting endometrial repair. However, effective clinical interventions based on these mechanisms remain largely unexplored.

Recent advances in immunotherapy have provided new insights into potential treatments for fibrotic diseases. CAR T‐cell therapy, which has revolutionized cancer treatment (Qian et al. 2024), is now being explored for applications beyond oncology, including autoimmune (Chung et al. 2024) and fibrotic diseases (Rurik et al. 2022). Here, we employed previously established CAR T‐cell repertoire technology with a diversity of 10^6^ to explore potential therapeutic strategies for IUA, and we successfully validated fibroblast activation protein (FAP) as a key target involved in the fibrotic process. Our data further suggested that T‐cell‐based therapeutics, such as the CAR‐T‐cell repertoire or FAP‐directed CAR‐T‐cell therapy, exerted potent anti‐fibrotic effects by targeting FAP‐positive cells, but at the cost of severe systemic toxicity, which led to cytokine release syndrome and high mortality rates in animal models. Given these challenges, we sought to develop a safer, cell‐free alternative that could retain the therapeutic benefits of CAR T cells while minimizing their associated toxicity. To this end, we leveraged a FAP‐targeting CAR‐T‐cell‐derived small extracellular vesicle (sEV) platform, which allowed us to selectively deliver FAP‐targeting therapeutic cargo in a more controlled manner. Furthermore, functional assessments suggested that this approach preserved reproductive potential, highlighting its translational value for restoring fertility in IUA patients.

## 2 Results

### 2.1 CAR Clone Shows Both a Therapeutic Effect and High Toxicity in a Model of Intrauterine Adhesion

**FIGURE 1:**
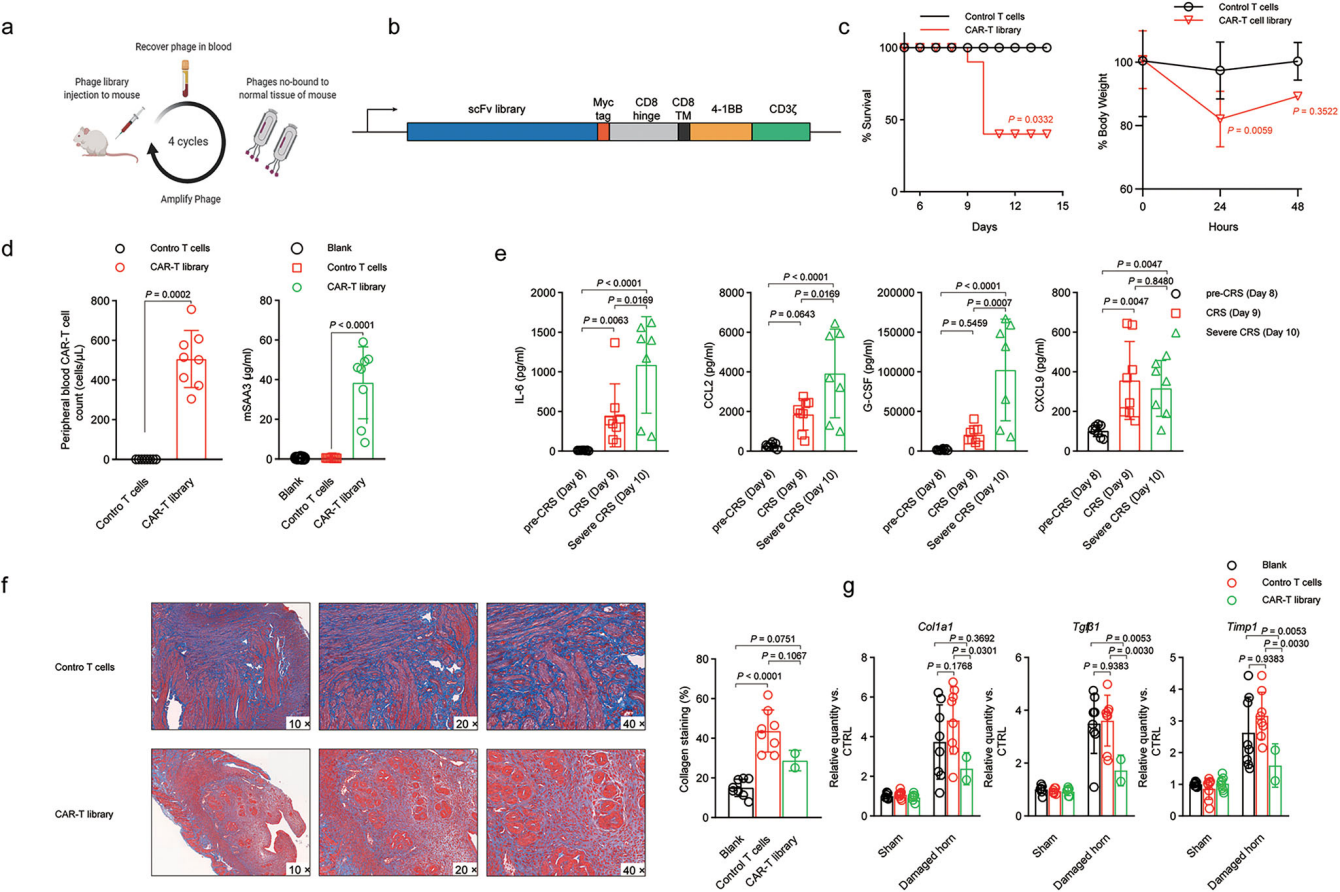
**The CAR‐T‐cell library improved regeneration of the damaged endometrium but was associated with a high risk of cytokine release syndrome**. (a) Negative selection: In vivo phage display was performed to collect phages that did not bind to normal tissues in mice. (b) Map of lentiviral constructs encoding the CAR library. (c) Percentage change in body weight in IUA mice and percentage survival of IUA mice in different treatment groups. (d) Left: peripheral blood was collected, and the absolute number of CAR T cells per microlitre of blood was quantified. Right: serum levels of murine SAA3 measured at the indicated time points. (e) Serum levels of mouse cytokines measured at the indicated time points. (f) Masson's trichrome staining was used to evaluate collagen deposition (blue) in the treated uterine horns of IUA model mice. (g) qPCR analyses of the expression of fibrosis‐related factors in the treated uterine horns of IUA model mice. The data are presented as the means ± SDs (c, d, e, f, g), and the p values are from the log‐rank test (Mantel–Cox) (c) a two‐sided nonparametric t test (d‐left), one‐way ANOVA followed by Tukey's post hoc test (d‐right, e, f) and two‐way ANOVA followed by the Bonferroni post hoc correction (g).

We first constructed a naïve synthetic cell library following previously established methodologies (Fu et al. 2022). This library was designed to facilitate the display of a diverse CAR repertoire on T cells. Specifically, the CAR constructs incorporated single‐chain variable fragments (scFvs) fused to a CD8α hinge, a transmembrane domain, and the intracellular signalling domains of human 4‐1BB and CD3ζ (or z), a configuration consistent with second‐generation CAR‐T‐cell designs. To ensure broad and unbiased scFv diversity, we collected peripheral blood samples from 200 non‐immunized, healthy volunteers to construct a naïve, non‐immunized human scFv library. Complementary DNA encoding the variable regions of the immunoglobulin heavy and light chains was synthesized from B lymphocytes and subsequently used to generate a phage‐displayed scFv library comprising approximately 1 × 10^8^ unique clones. A quality assessment of the library revealed that over 98% of the clones contained a correctly sized scFv insert, confirming the integrity of the constructed repertoire. To further assess the heterogeneity and diversity of the scFv library, we performed sequencing analysis on 50 randomly selected clones. Each clone exhibited a distinct scFv sequence, demonstrating the library's vast antigen recognition potential. However, since human‐derived phage display libraries may contain scFvs that cross‐react with murine tissues, we implemented an in vivo negative selection strategy to eliminate phages that bound non‐specifically to mouse systemic organs. This process involved four rounds of in vivo biopanning in BALB/c mice, allowing the selective enrichment of phages that exhibited minimal off‐target binding (Figure 1a). Following this negative selection step, the DNA sequences of the remaining isolates were analysed, confirming that library diversity was maintained. This refined phage pool was then stochastically downsampled and used to generate a CAR‐T‐cell library containing approximately 2 × 10^6^ unique CAR constructs (Figure 1b). The resulting CAR‐T‐cell library provides a comprehensive and diverse set of antigen‐binding receptors, offering a valuable platform for functional screening and therapeutic development.

Following the protocol described by Alawadhi et al. (Alawadhi et al. 2014), we established a mouse model of AS and generated a diverse CAR‐T‐cell library for adoptive transfer immediately after uterine incision in anaesthetized mice. In our initial trials, we observed that the engraftment efficiency of donor T cells was suboptimal. To enhance the in vivo persistence and proliferation of the transferred cells, we preconditioned the mice with cyclophosphamide to achieve effective lymphodepletion prior to CAR‐T‐cell administration. Unexpectedly, infusion of the CAR‐T‐cell library triggered an acute inflammatory response in IUA model mice. By Day 9 after transfer, the treated animals exhibited significant clinical signs of distress, including diminished activity, generalized malaise, piloerection, and marked weight loss. These adverse reactions culminated in high mortality rates among the treated cohort (Figure 1c). Biochemical analyses revealed a pronounced increase in serum amyloid A3 (SAA3), the murine homologue of C‐reactive protein (CRP), which is commonly elevated during inflammatory events in clinical settings (Davila et al. 2014, Lee et al. 2015); this finding further confirmed the occurrence of a cytokine release syndrome (CRS)‐like event (Figure 1d). Additionally, key pro‐inflammatory mediators, such as IL‐6, CCL2, G‐CSF, and CXCL9, were significantly upregulated (Figure 1e), and their levels strongly correlated with the severity of CRS and overall survival outcomes. These data collectively indicate that the CAR‐T‐cell library induced robust CRS in the IUA mouse model.

In contrast to the severe toxicity observed in the majority of the animals, two out of eight mice that received the CAR‐T‐cell library experienced transient toxicity characterized by rapid weight loss but eventually recovered from the acute phase of inflammation. One week after the resolution of these toxic effects, we performed a detailed histopathological evaluation of the uterine tissues from these surviving mice. Masson's trichrome (MT) staining revealed that the CAR‐T‐cell library markedly reduced the extent of fibrotic lesions in the damaged uteri, indicating a significant attenuation of fibrosis (Figure 1f). Moreover, quantitative analyses of key fibrosis‐related markers further supported these histological findings. Specifically, the expression levels of Col1a1, Timp1, and transforming growth factor beta 1 (Tgfb1) were substantially decreased in uterine tissues following CAR‐T‐cell administration (Figure 1g). These molecular changes suggest that the CAR‐T‐cell library ameliorated the pathological fibrotic condition associated with AS.

In summary, although the CAR‐T‐cell library induced severe CRS that resulted in high mortality, the surviving animals demonstrated significant therapeutic benefits. The treatment not only reduced uterine fibrosis, as evidenced by MT staining and improved histopathological scores, but also normalized the expression of critical molecular markers involved in fibrosis. These findings highlight both the potential efficacy and the challenges associated with CAR‐T‐cell therapies in treating fibrotic uterine disorders, emphasizing the need for further optimization to mitigate adverse inflammatory responses.

### 2.2 Identification of the Antigen Bound by C21D5 as FAP

**FIGURE 2:**
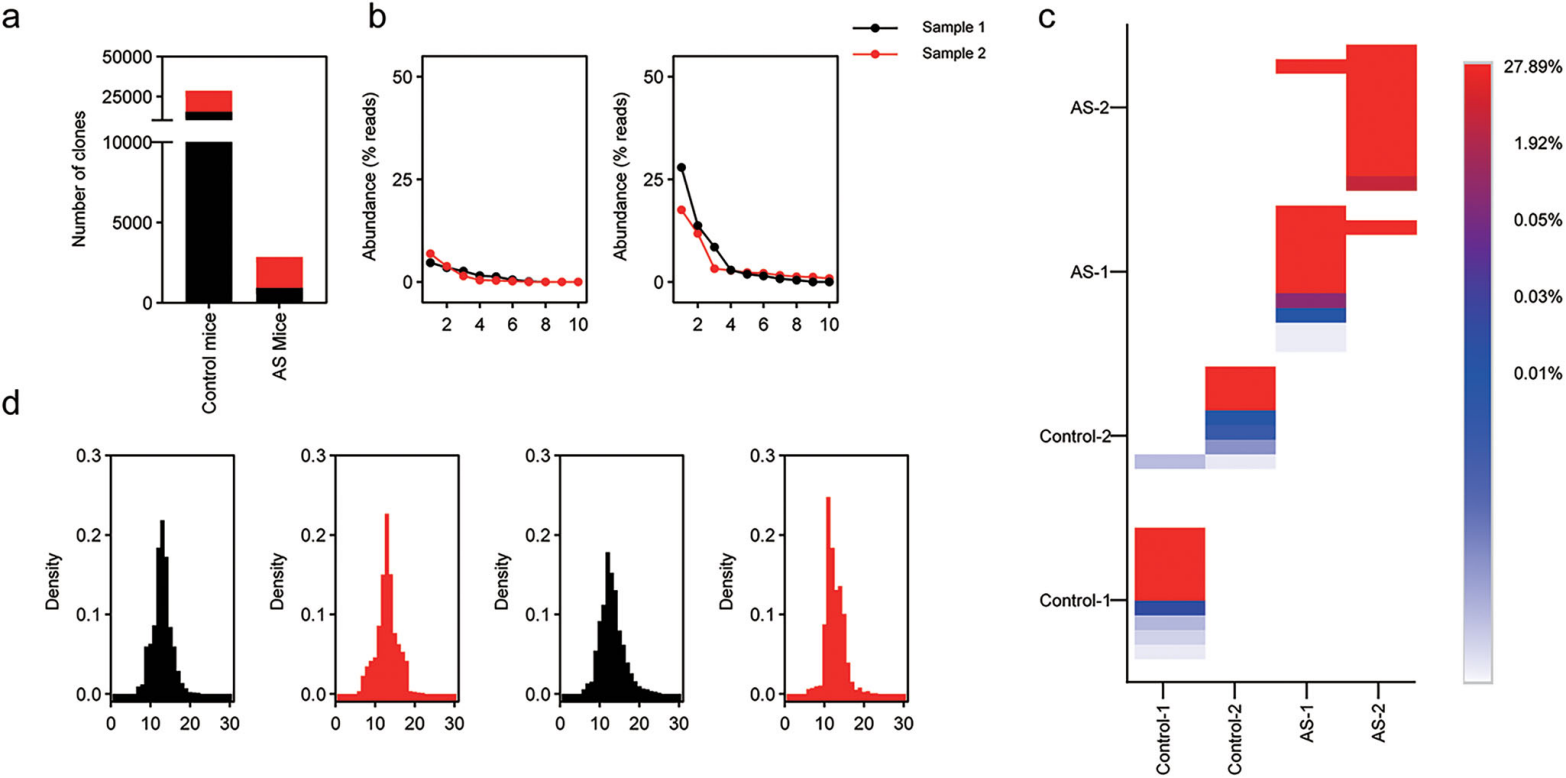
**CAR diversity in IUA models**. (a) The number of unique scFv clones derived from CAR+ cells sorted from different animal models. (b) Oligoclonality of the various libraries represented by the top ten most abundant clones in each library. The *x*‐axis displays the clones in decreasing abundance, whereas the *y*‐axis represents the abundance (as a percentage of sequencing reads) of the corresponding clones. (c) Comparison of high‐frequency heavy chain CDR3s (CDRH3s) reveals unique VH genes in each mouse treated with the CAR library. Heatmap showing the distribution of highly represented CDRH3s in different animals. The *y*‐axis represents the ten most frequent CDRH3 sequences identified in each mouse. The *x*‐axis compares the frequency of these prevalent CDRH3 sequences across the other mice. (d) Density plots of the distribution of CDRH3 amino acid lengths of the scFv clones for the various libraries.

To comprehensively characterize the CAR repertoires enriched in vivo, we performed deep sequencing of CAR constructs recovered from CAR‐positive T cells isolated from two surviving IUA mice. As controls, CAR‐positive T cells were similarly isolated from two sham‐operated mice that had received the CAR‐T‐cell library for 14 days. In the IUA model, each mouse yielded a comparable number of unique CAR clones (range: 951–1927). However, the AS‐enriched libraries retained only approximately 10–20% of the clonal diversity observed in the control libraries (Figure 2a), indicating substantial clonal contraction under fibrotic conditions and suggesting strong in vivo selection pressure within the injured uterine microenvironment.

To assess clonal dominance, we quantified the abundance of individual CAR sequences based on their respective sequencing read counts. In control CAR libraries, oligoclonality was minimal, with fewer than 0.1% of clones exceeding 1% of total sequencing reads. In contrast, AS‐enriched libraries exhibited a pronounced oligoclonal profile, with approximately 10 clones accounting for a substantial proportion of total reads in each mouse. In the most extreme case, a single clone represented 27.9% of total sequencing reads (Figure 2b), demonstrating strong selective expansion. Although the overall CAR repertoires differed between genetically identical littermates, reflecting host‐specific evolutionary dynamics, one specific CAR sequence was reproducibly enriched at high frequency in both independent AS mice but was not enriched in control libraries (Figure 2c). This convergence across independent biological replicates indicates that, despite broader repertoire divergence, common selective pressures operate within the fibrotic microenvironment. This recurrently enriched clone was designated C21D5 and selected for further investigation. Other expanded clones were detected within individual AS mice; however, these did not demonstrate reproducible enrichment across animals, suggesting that their expansion may reflect stochastic or host‐specific factors rather than shared disease‐associated antigen recognition.

To further explore structural features associated with selection, we analysed the distribution of complementarity‐determining region 3 of the heavy chain (CDRH3) lengths. Control libraries exhibited a near‐normal distribution centred at a median length of 14 amino acids. In contrast, AS‐enriched libraries showed a shift toward shorter CDRH3 sequences, with a median length of 11 amino acids (Figure 2d). This contraction in CDRH3 length distribution is consistent with selective pressure favouring specific antigen‐binding geometries within the IUA microenvironment. Collectively, these deep sequencing analyses reveal marked differences in clonal diversity, oligoclonal expansion, and structural repertoire characteristics between AS and control conditions. Importantly, despite individual variability, the reproducible enrichment of C21D5 across independent AS mice suggests the presence of a shared disease‐associated antigen driving CAR selection.

To identify the antigen bound by C21D5, we employed a multistep approach integrating biochemical and biophysical methods. First, we biotinylated uterine cell surface proteins and performed immunoprecipitation (IP) using matrix‐immobilized C21D5 scFv‐Fc, as depicted in Figure 1a (Asensio et al. 2019). The immunoprecipitated proteins were then digested and subjected to tandem mass spectrometry analysis, which identified the target protein as mouse FAP. To further validate this finding, we conducted ectopic expression and flow cytometry assays. HEK293 cells were transiently transfected with a FAP expression plasmid to generate 293‐FAP cells. In parallel, we produced a recombinant C21D5 IgG1 antibody. Flow cytometry analysis demonstrated that C21D5 IgG1 specifically bound to 293‐FAP cells, whereas no binding was detected in the parental HEK293 cells (Figure S1). This specificity confirmed that the target of C21D5 is indeed FAP. Next, surface plasmon resonance (SPR) was used to quantify the monovalent affinity of C21D5 for FAP. The SPR results revealed that the binding affinities of C21D5 for FAP were comparable to those reported for other anti‐FAP antibodies (Table S1). Together, these experiments provide strong, multi‐faceted evidence that FAP is the antigen recognized by C21D5, underscoring its potential relevance in targeted therapeutic applications.

### 2.3 FAP^+^ Cells Are Highly Abundant In Both The IUA Mouse Model and Human Specimens

**FIGURE 3:**
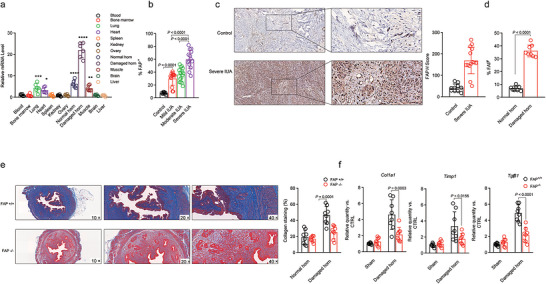
**FAP is a crucial factor in IUA**. (a) qPCR analysis of the expression of FAP mRNA in different tissues of a mouse model normalized to Gapdh; values are relative units. The data are from four independent biological replicates. (b) FAP expression in cells from endometrial tissue samples was measured by flow cytometry. (c) Immunohistochemical staining of FAP in endometrial tissue samples. Scale bars, 100 mm. The results are expressed as the means ± SEMs. (d) FAP expression in cells from endometrial tissue samples was measured by flow cytometry. (e) Masson's trichrome staining was used to evaluate collagen deposition (blue) in treated uterine horns of IUA FAP^+/+^ or FAP^−/−^ mice. (f) qPCR analyses of the expression of fibrosis‐related factors in the treated uterine horns of IUA FAP^+/+^ or FAP^−/−^ mice. The data are presented as the means ± SDs (a–f), and the *p* values are from one‐way ANOVA followed by Tukey's post hoc test (a, b), a two‐sided nonparametric t test (c, d), and two‐way ANOVA followed by the Bonferroni post hoc correction (e, f). *P < 0.05; **P<0.01; ***P < 0.001; ****P < 0.0001 (a).

To elucidate the role of FAP in IUA), we first evaluated FAP expression in an established IUA mouse model. All organs were harvested from euthanized mice, weighed, and analysed for FAP RNA expression. Remarkably, a significant upregulation of FAP was observed in the uterine horn affected by AS, whereas the normal horn and other tissues presented only low levels of expression (Figure 3a). In parallel, we examined FAP protein expression in human subjects. We included 43 patients with intrauterine adhesions, with the average patient age being 29.5 years. According to the American Fertility Society (AFS) criteria, 23% of these patients were classified as having severe IUA (score ≥ 9 points), and 37% were classified as having moderate IUA (score 5–8 points). Endometrial biopsies were collected from these patients, and the frequency of FAP‐positive cells was quantified using flow cytometry analysis. Notably, patients with severe IUA presented a significantly greater number of FAP‐positive cells than did those with moderate or mild disease (Figure 3b). Immunohistochemical analysis also confirmed that FAP protein levels were notably increased in IUA patients (Figure 3c). Similarly, in the IUA mouse model, the damaged uterine horn contained greater numbers of FAP‐positive cells than did the normal horn (Figure 3d). To investigate the functional role of FAP in fibrosis and adhesion formation, we generated homozygous FAP knockout (FAP^–/–^) mice. Histopathological analysis using Masson's trichrome staining revealed a marked reduction in the severity of intrauterine adhesions in the damaged uterine horn of FAP^–/–^ mice compared with their FAP^+/+^ counterparts (Figure 3e). Furthermore, quantitative analyses demonstrated that the expression of key fibrosis‐related genes—such as Col1a1, Timp1, and Tgfb1—was significantly downregulated in the injured uterine tissue of FAP^–/–^ mice (Figure 3f). Collectively, these findings suggest that FAP not only constitutes a biomarker for IUA but also plays a pivotal role in mediating fibrosis‐associated adhesion. Elevated FAP expression is correlated with disease severity, and its absence significantly mitigates fibrotic damage, highlighting FAP as a potential therapeutic target in IUA management.

### 2.4 FAP‐Targeting T Cells Have Both Therapeutic Effects and Toxic Effects

**FIGURE 4:**
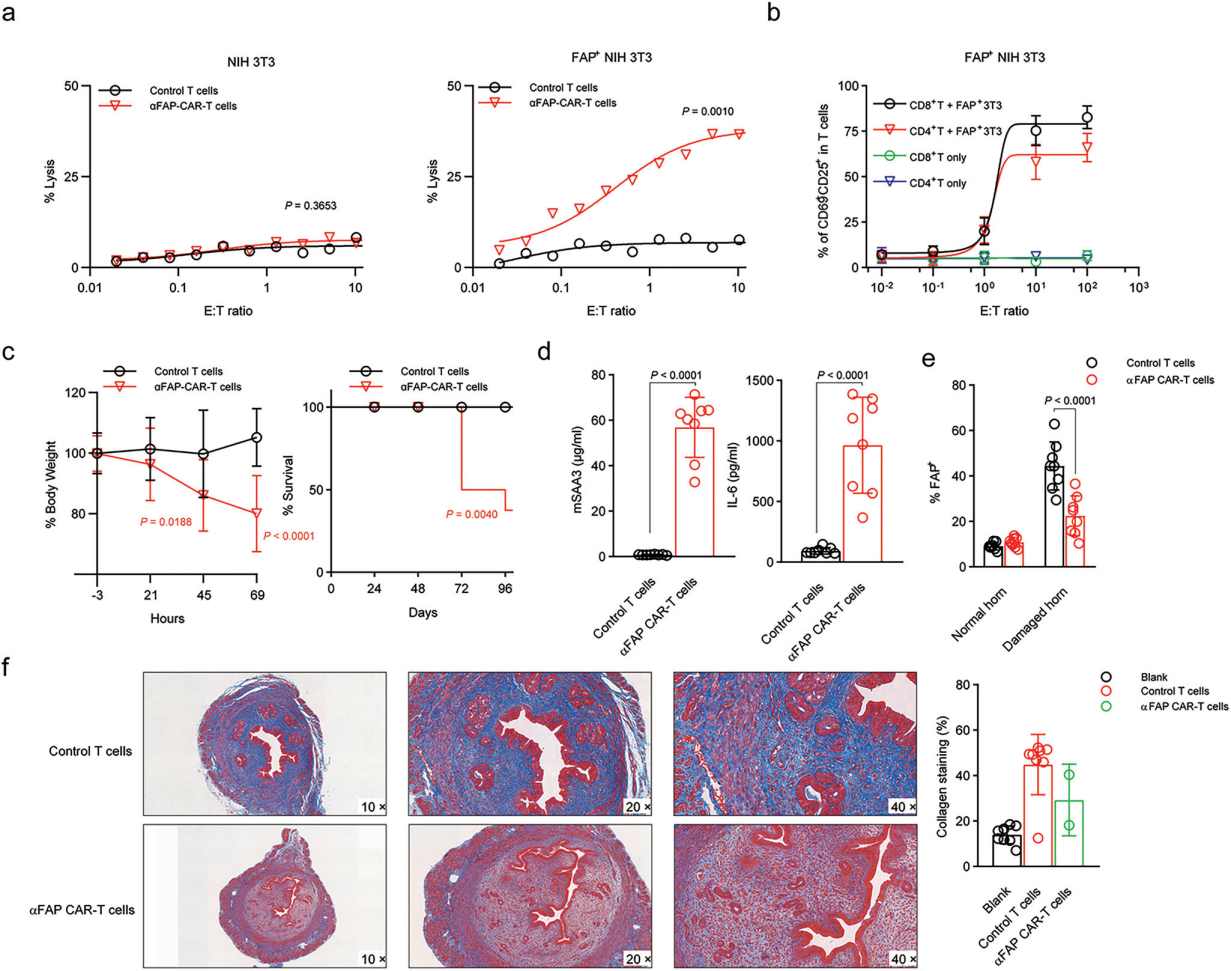
**FAP‐targeting T cells had both therapeutic effects and toxic effects in an IUA model**. (a) Killing activity of CAR T cells in response to target cells. The cytotoxic activity of CAR T cells and control T cells against different cell lines was assessed by a 51Cr release assay at the indicated effector‐to‐target (E:T) ratios. (b) FAP+ 3T3 cells and purified CD8+ CAR T cells or CD4+ CAR T cells were incubated for 24 h. The ratio of T cells to target cells was 5:1. Activated T cells, marked as CD69+CD25+ cells, were measured and calculated. (c) Percentage change in body weight and percentage survival of IUA mice in different treatment groups. (d) Serum levels of murine SAA3 and IL‐6 were measured at the indicated time points. (e) FAP expression in cells from endometrial tissue samples was measured by flow cytometry. (f) Masson's trichrome staining was used to evaluate collagen deposition (blue) in the uterine horns of IUA mice subjected to different treatments. The data are presented as the means ± SDs (a–f), and the p values are from a nonparametric t test (a, d), two‐way ANOVA followed by the Bonferroni post hoc correction (b, c, e), and the log‐rank test (Mantel–Cox) (c).

Next, we investigated the anti‐IUA potential and specificity of C21D5‐transduced CAR T cells using standard 51Cr release assays. In these experiments, we employed both parental 3T3 cells and genetically engineered 3T3‐FAP cells that expressed FAP. Our results demonstrated that CAR T cells expressing the C21D5 single‐chain variable fragment (scFv) selectively lysed FAP‐positive 3T3‐FAP cells while sparing parental 3T3 cells (Figure 4a). In addition, when purified CD4+ and CD8+ CAR T cells were separately used as effectors, both subsets were activated to similar extents, as evidenced by upregulated expression of the activation markers CD69 and CD25 (Figure 4b). Importantly, T‐cell activation was strictly dependent on the presence of FAP‐positive target cells, underscoring the specificity of the C21D5 CAR construct.

To evaluate whether monoclonal C21D5 CAR T cells could exert therapeutic effects in vivo while minimizing side effects, we adoptively transferred these cells directly into AS mice. Unfortunately, within 24 h after transfer, the treated animals exhibited signs of CRS, including reduced mobility, rapid weight loss, and other systemic toxicities. This toxicity was fatal in the majority of cases, with five out of eight animals succumbing within 72 h (Figure 4c). Serial monitoring of blood samples revealed significant elevations in murine SAA3 and IL‐6 levels (Figure 4d), which are hallmarks of an acute inflammatory response. Notably, three of eight animals exhibited transient toxicity, ultimately recovered, and were re‐evaluated two weeks later. Interestingly, CAR‐T‐cell therapy notably reduced the frequency of FAP‐positive cells in the damaged uterine horn (Figure 4e). Histological analysis using Masson's trichrome staining revealed that C21D5 CAR‐T‐cell treatment led to a marked reduction in fibrotic lesions in the damaged uteri of these survivors, suggesting that immune intervention targeting FAP‐positive cells can effectively inhibit the progression of intrauterine adhesion (Figure 4f).

**FIGURE 5:**
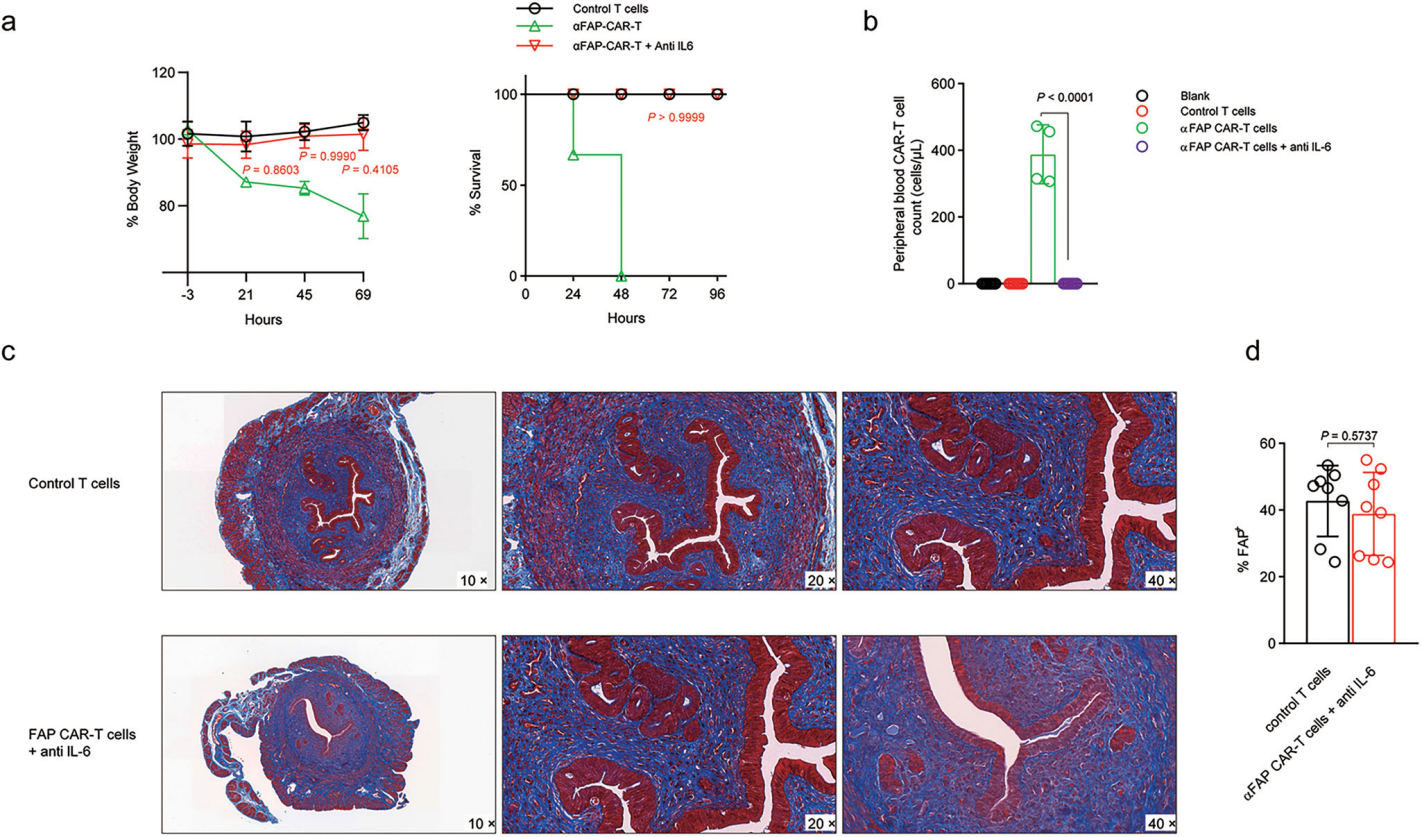
**Combining FAP‐targeting T cells with IL‐6 blockade had no therapeutic benefit**. (a) Percent change in body weight in IUA mice and percent survival of IUA mice in different treatment groups. (b) Peripheral blood was collected, and the absolute number of CAR‐T cells per microlitre of blood was quantified.(c) Masson's trichrome staining was used to evaluate collagen deposition (blue) in the uterine horns of IUA mice subjected to different treatments. (d) FAP expression in cells from endometrial tissue samples was measured by flow cytometry. The data are presented as the means ± SDs (a−d), and the *p* values are from a nonparametric t test (a, d) and one‐way ANOVA followed by Tukey's post hoc test (b).

Given the concerning levels of toxicity observed with CAR‐T‐cell therapy in this model, we explored the potential of mitigating these adverse effects by concurrently administering an IL‐6 blockade. When adoptive cell therapy was combined with IL‐6 inhibition, the treated mice did not exhibit the previously observed weight loss or mortality, indicating that the IL‐6 blockade successfully prevented CRS‐associated toxicity (Figure 5a). However, peripheral blood analysis indicated that the engineered T cells failed to expand in vivo under IL‐6 blockade conditions (Figure 5b). Consequently, further evaluation at two weeks after treatment revealed that the combination therapy did not yield any significant therapeutic benefit, as the number of FAP‐positive cells as well as the extent of Masson's trichrome staining in the uterine tissue remained comparable to what was observed in the control group (Figure 5c).

Collectively, these findings highlight the complex balance between therapeutic efficacy and safety in CAR‐T‐cell‐based interventions for intrauterine adhesion. While C21D5 CAR T cells demonstrate potent and specific cytotoxicity against FAP‐positive targets and can reduce fibrosis in damaged uterine tissue, their application is limited by severe CRS‐induced toxicity. Moreover, attempts to mitigate toxicity through IL‐6 blockade, although effective in reducing adverse reactions, have also impeded the in vivo expansion and anti‐IUA activity of CAR T cells, ultimately negating their therapeutic benefit. These results underscore the need for further optimization of CAR‐T‐cell strategies to achieve both safety and efficacy in treating fibrotic disorders such as IUA.

### 2.5 CAR Exosomes Reverse Endometrial Fibrosis and Restore the Pregnancy Capability of the IUA Model in Mice

Previously, we established a method for enriching sEVs derived from CAR T cells. CAR‐T‐cell‐derived sEVs have been demonstrated to be alternative effectors for CAR‐mediated antigen specificity and cytotoxicity. CAR‐containing sEVs can also express high levels of cytotoxic molecules, recognizing target antigens and mediates antigen‐specific cytotoxicity. Importantly, no CRS‐related toxicity was observed during treatment in preclinical xenograft models, indicating the efficiency and safety of these therapeutic agents. Therefore, this method enables the efficient isolation and characterization of CAR‐T‐derived sEVs, providing a potential cell‐free therapeutic approach for targeting intrauterine adhesion.

**FIGURE 6:**
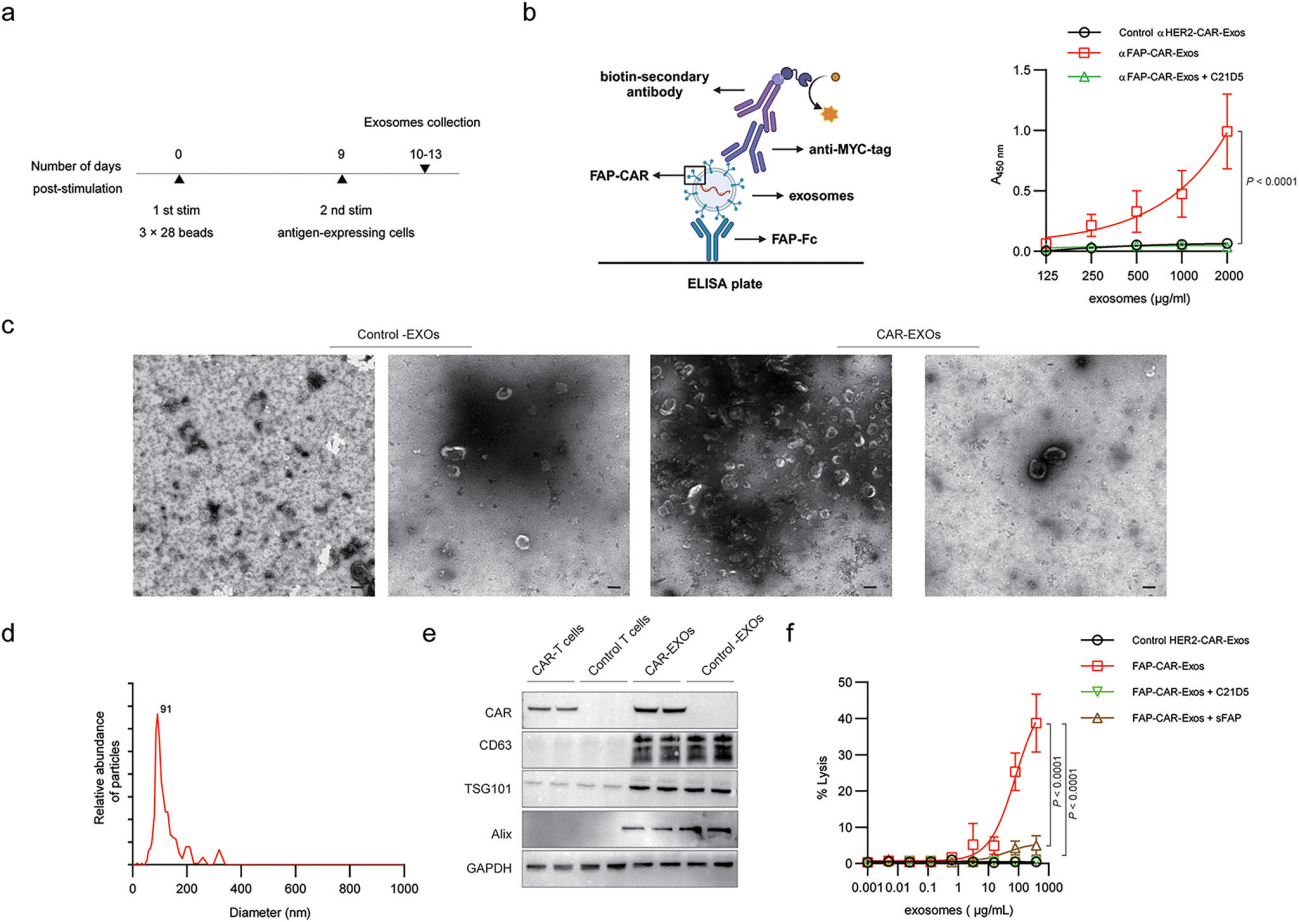
**Characterization of FAP‐targeting extracellular vesicles**. (a) Schematic representation of the enrichment of CAR‐containing exosomes from T cells with repeated antigen stimulation. (b) Antigen binding by exosomes with or without blocking antibodies. (c) Transmission electron micrographs of CAR exosomes. The samples were negatively stained with uranyl acetate. Scale bars = 250 nm or 100 nm. (d) Size distribution of CAR exosomes as measured by NTA, peaking at a 91‐nm diameter. (e) Immunoblots for CAR expression in whole‐cell lysates and purified exosomes from CAR‐T cells. (f) Killing activity of CAR exosomes in response to target cells. The cytotoxic activity of CAR exosomes against target cell lines was assessed by the ^51^Cr release assay at the indicated concentrations. The data are presented as the means ± SDs, and the p values are from two‐way ANOVA followed by the Bonferroni post hoc correction (b, f).

We stimulated C21D5 CAR T cells with a previously described two‐stage strategy over the course of 2 weeks in vitro to isolate CAR exosomes (Figure 6a). T‐cell surface CARs can bind to antigens through their extracellular domain to achieve a targeting effect. Analysis by enzyme‐linked immunosorbent assay (ELISA) and Western blotting revealed exosomal CAR has the same membrane topology as cell surface CAR, with its extracellular domain exposed on the surface of the exosomes. The exosomal CAR binds antigens in a concentration‐dependent manner, and this interaction can be disrupted by blocking antibodies. The exosomes produced were physically homogeneous, with a size distribution peaking at an 80‐nm diameter, as determined by nanoparticle tracking analysis (NTA) and electron microscopy (Figure 6b–e). We also investigated their cytotoxic effects on antigen‐expressing cell lines and exosomes from HER2‐targeting CAR T cells, which served as the control (Fu et al. 2019) (Figure 6f). αFAP‐CAR exosomes induced dose‐dependent cytotoxicity against FAP‐expressing target cells, whereas exosomes derived from control CAR T cells showed no detectable activity. Cytotoxicity mediated by αFAP‐CAR exosomes was partially attenuated by the FAP‐blocking antibody C21D5 and by soluble FAP (sFAP) competition, supporting a CAR‐defined, target‐dependent mode of action. Antibody blockade is more efficient than soluble ligand competition likely due to avidity and local concentration at membrane‐bound FAP. To examine whether CAR exosome‐induced cytotoxicity involves apoptotic pathways, target cells were pretreated with the pan‐caspase inhibitor Z‐VAD‐FMK prior to exosome exposure. Z‐VAD‐FMK significantly reduced αFAP‐CAR exosome‐induced target cell lysis across multiple concentrations, whereas Z‐VAD‐FMK had no effect on basal viability in the presence of control HER2‐CAR exosomes (Figure S2a). We next compared two independent αFAP‐CAR exosome preparations with differential granzyme B content. Consistent with their granzyme B levels, the GzmB^high^ exosome preparation induced greater target cell lysis than the GzmB^low^ preparation across matched concentrations (Figures S2b and S2c).

Having established antigen specificity and granzyme‐dependent cytotoxicity in vitro, we next investigated the in vivo behaviour and therapeutic efficacy of CAR exosomes in the IUA model. To evaluate tissue distribution under pathological conditions, CAR exosomes were administered intravenously following uterine injury induction. CAR‐associated signals were detectable in injured uterine tissue during the early post‐injection period and declined over time, consistent with transient biodistribution and progressive clearance (Figure S3a). Importantly, repeated administration did not result in significant alterations in body weight, serum biochemical parameters, or histopathological abnormalities in major organs (Figure S3b‐d), indicating a favourable short‐term safety profile in the IUA model.

**FIGURE 7:**
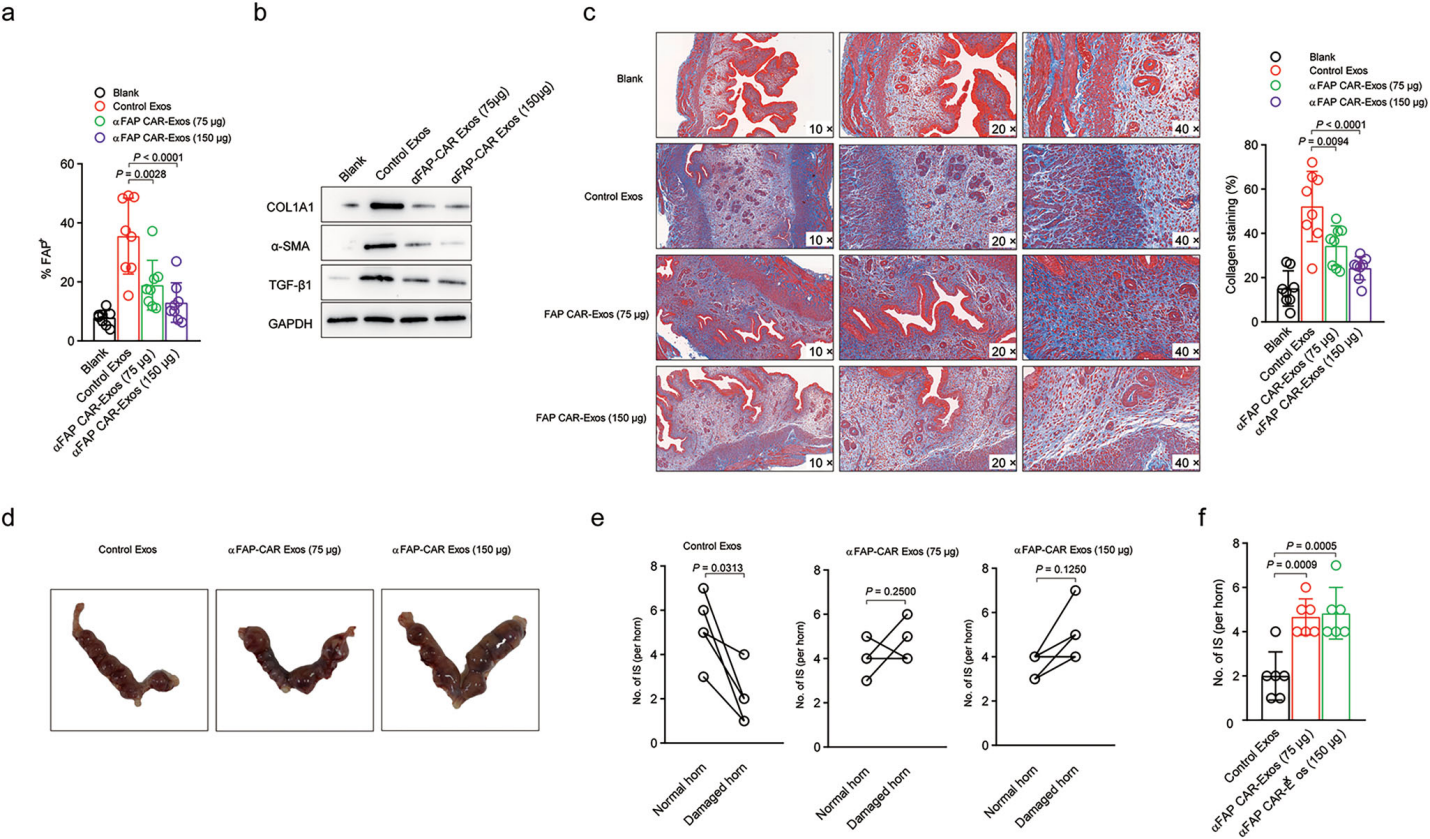
**FAP‐targeting EVs improve regeneration of the damaged endometrium and is associated with better implantation outcomes**. (a) FAP expression in cells from endometrial tissue samples was measured by flow cytometry. (b) Immunoblots for COL1A1, α‐SMA, and TGF‐β1 proteins in damaged IUA uteri with different treatments. GAPDH was used as a loading control. (c) Masson's trichrome staining was used to evaluate collagen deposition (blue) in the treated uterine horns of IUA mice subjected to different treatments. (d) Gross morphology of the implantation sites (ISs) in IUA mice subjected to different treatments. (e) The numbers of ISs in different uterine horns of mice subjected to different treatments. (f) The numbers of ISs in the damaged uterine horns of mice subjected to different treatments. Doses are expressed as µg protein per mouse. The data are presented as the means ± SDs (a, c, f), and the *p* values are from one‐way ANOVA followed by Tukey's post hoc test (a, c, f) and a paired nonparametric *t* test (e).

We then assessed antifibrotic efficacy based on the unique biological functions of sEVs derived from CAR T cells. In this study, we first established a treatment protocol by administering multiple doses of sEVs over a two‐week period concurrently with the induction of the IUA model. The rationale behind this strategy was to provide continuous immunomodulatory support during the early stages of endometrial injury, thereby preventing the progression of fibrotic processes. Following the completion of the treatment regimen, comprehensive endometrial tissue analyses were conducted. Compared with that in control mice, a notable reduction in the number of FAP‐positive cells in the uterine tissue of sEV‐treated mice was detected (Figure 7a). The expression levels of critical fibrosis markers, including type I collagen (COL1A1), α‐smooth muscle actin (α‐SMA), and transforming growth factor beta 1 (TGF‐β1), were further evaluated. The data revealed that exosome administration led to downregulation of COL1A1, α‐SMA, and TGF‐β1 expression in the uterine tissue on the model side, corroborating the evidence of reduced FAP+ cells. (Figure 7b). Moreover, Masson's trichrome staining revealed a marked decrease in both the extent and area of fibrosis in endometrial tissue following exosome therapy (Figure 7c).

At 14 days after IUA induction, male and female mice were co‐housed to allow natural mating, with the detection of a vaginal plug designated embryonic Day 0.5. On embryonic Day 8, the mice were sacrificed to evaluate reproductive outcomes. The numbers of embryo implantation sites in both the treated and control uterine horns were recorded, and differences in embryo size and uterine morphology were carefully observed. In control EV–treated mice, a reduction in embryo implantation in the injured uterine horn was noted compared with that on the control side, with some mice showing no implantation at all (Figure 7d). In contrast, exosome‐treated mice exhibited embryo implantation on the injured side that was comparable to that on the control side, suggesting that exosome therapy can significantly increase the implantation rate in the damaged uterus.

On embryonic Day 12, another cohort of mice was sacrificed to further assess embryo and placental development. The embryos and placentas were carefully dissected and evaluated (Figure 7e‐f). In control EV–treated mice, the injured uterine horn harboured embryos with developmental delays and growth arrest, along with correspondingly underdeveloped placentas. In contrast, embryos and placentas from the exosome‐treated group exhibited normal growth and morphology (Figure S4a). Subsequent skeletal staining of normally developing embryos from both the control EV–treated and CAR exosome‐treated groups revealed well‐formed bone structures, with no signs of skeletal malformation in either group (Figure S4b).

Collectively, these results suggest that uterine endometrial damage primarily compromises the early stage of embryo implantation, thereby reducing the implantation rate. However, once successful implantation occurs, subsequent embryonic development proceeds normally with proper placental support. Exosome treatment appears to mitigate endometrial fibrosis, thus increasing implantation efficiency in the early stages of pregnancy, and ultimately restores reproductive function from impairment by intrauterine adhesions. Overall, our data provide compelling evidence that sEV‐based therapy can mitigate the development of intrauterine adhesions by reducing both the cellular and molecular hallmarks of endometrial fibrosis. This approach offers a promising cell‐free therapeutic strategy that could overcome the limitations associated with direct cell‐based therapies, including the risk of severe toxicity and adverse immune reactions.

## 3 Discussion

IUA, or AS, remains a significant clinical challenge, particularly in reproductive medicine. IUA is characterized by the formation of fibrotic scar tissue within the uterine cavity, which can lead to menstrual abnormalities, infertility, and recurrent pregnancy loss. Current therapeutic strategies, primarily surgical adhesiolysis followed by hormonal therapy, are often associated with high recurrence rates and limited efficacy, particularly in severe cases. These drawbacks underscore the urgent need for novel therapeutic approaches that target the underlying pathophysiology of IUA rather than merely addressing its manifestations.

Recent advances in immunotherapy have opened new avenues for the treatment of fibrotic diseases, including IUA. In this study, we explored the feasibility of targeting FAP–positive cells as a means to modulate fibrotic remodelling in the uterus. Although FAP has been implicated in fibrotic processes, its suitability as a therapeutic target requires careful consideration of spatial and contextual expression patterns. In our IUA model, FAP expression was predominantly detected in the injured uterine horn, while expression in other examined tissues remained low or near background. Similarly, analysis of human endometrial samples revealed a disease‐severity–dependent enrichment of FAP‐positive cells, with relatively low baseline expression in non‐fibrotic endometrium. Functional evidence from FAP‐deficient mice further supports a contributory role of FAP‐positive fibroblasts in intrauterine fibrosis. Nevertheless, FAP is not an absolutely tissue‐restricted marker and may be transiently expressed in other contexts involving tissue injury or repair. These considerations highlight the importance of intervention modality and dosing strategy when targeting FAP in fibrotic remodelling. In this regard, the observed toxicity of FAP‐directed CAR T cells underscores the limitations of cell‐based approaches in fibrotic diseases, whereas the EV‐based strategy offers a more controlled means of engaging disease‐associated FAP‐positive cells.

In vitro cytotoxicity assays confirmed that C21D5‐transduced CAR T cells selectively lysed FAP‐expressing cells while sparing cells lacking FAP expression. This antigen specificity is crucial for the development of safe and effective immunotherapies. However, the translation of these findings into in vivo models revealed significant challenges. The adoptive transfer of monoclonal C21D5 CAR T cells into IUA mice induced pronounced CRS, characterized by systemic inflammation, weight loss, and reduced mobility, ultimately resulting in high mortality rates. The markedly elevated levels of murine SAA3, IL‐6, and other pro‐inflammatory cytokines mirrored the clinical manifestations of CRS observed in patients receiving CAR‐T‐cell therapy for malignancies. This severe toxicity underscores the potential risks of using CAR T cells in non‐malignant fibrotic conditions such as IUA, where the balance between the therapeutic benefit and the systemic inflammatory response is particularly precarious.

In this study, we observed that pharmacological blockade of IL‐6 signalling effectively prevented CRS induced by FAP‐targeting CAR‐T cells, but was also associated with a marked impairment of in vivo CAR‐T expansion and persistence. Under IL‐6 blockade conditions, circulating CAR‐T cells were barely detectable, and this loss of expansion coincided with the absence of target cell clearance and antifibrotic efficacy. These findings suggest that, in this model, IL‐6 signalling is functionally linked to CAR‐T cell persistence and therapeutic activity (Barrett 2024). This observation differs from clinical experience with CD19‐directed CAR‐T therapies in hematologic malignancies, where IL‐6 receptor blockade is widely used to control CRS without substantially compromising CAR‐T expansion. We believe that this discrepancy reflects fundamental differences in biological context rather than a contradiction of established clinical paradigms. Intrauterine adhesion represents a non‐malignant, low‐antigen, non‐lymphoid tissue environment with limited inflammatory and antigen‐presenting support. In such settings, IL‐6 signalling may contribute not only to inflammatory toxicity but also to early CAR‐T survival and expansion.

Consistent with this notion, recent studies in non‐oncologic tissue injury models have highlighted that CAR‐T cell behaviour outside the tumour and lymphoid compartments is highly dependent on the local inflammatory milieu. Therefore, our data do not suggest a universal requirement for IL‐6 signalling in CAR‐T expansion, but instead indicate a context‐dependent role of IL‐6 in sustaining CAR‐T activity within fibrotic tissues. These findings highlight the context‐dependent nature of cytokine signalling in CAR‐T biology and caution against directly extrapolating cytokine‐management strategies established in hematologic malignancies to fibrotic or non‐malignant disease models. We have revised the manuscript to clarify this distinction and to avoid overgeneralization of these results beyond the present experimental setting.

In light of these challenges, our study also explored an alternative therapeutic modality: the use of sEVs derived from CAR T cells. Exosome‐based therapies offer several potential advantages over cell‐based therapies, including decreased immunogenicity, a reduced risk of systemic toxicity, and the ability to modulate cellular responses in a paracrine manner (Kalluri and McAndrews 2023). Our data demonstrated that exosome therapy significantly reduced fibrotic lesion burden in uterine tissue and partially improved uterine architecture during the early phase of fibrogenesis. The decrease in fibrosis was further corroborated by the downregulation of key fibrotic markers, such as Col1a1, Timp1, and Tgfb1, in exosome‐treated tissues. In addition to reducing the frequency of FAP‐positive fibroblasts, CAR‐EV treatment was also associated with decreased expression of α‐SMA and COL1A1 at the protein level and a marked reduction in collagen deposition as demonstrated by Masson staining. These coordinated changes suggest that the therapeutic effect of CAR‐EVs may extend beyond direct elimination of FAP‐positive cells.

FAP‐positive fibroblasts are not merely structural contributors to scar formation but are increasingly recognized as active regulators of extracellular matrix organization and profibrotic signalling networks. Previous studies have demonstrated that FAP‐expressing fibroblasts contribute to collagen deposition and TGF‐β–mediated fibrotic remodelling in multiple tissue contexts. Therefore, selective targeting of this cell population may have secondary consequences, including attenuation of TGF‐β–driven signalling cascades and partial normalization of matrix architecture (Kraman et al. 2010, Sahai et al. 2020). Taken together, our findings are consistent with a model in which CAR‐EVs contribute to microenvironmental remodelling within fibrotic uterine tissue through both direct targeting and indirect modulation of fibrotic signalling networks. While further mechanistic dissection will be required to distinguish direct cytotoxic from secondary remodelling effects, the coordinated reduction in fibroblast activation markers and collagen deposition supports a multifaceted mode of action.

Moreover, the therapeutic benefit of exosome treatment extended to improved reproductive outcomes. In our IUA mouse model, preventive exosome administration during model induction led to a significant increase in embryo implantation rates in the damaged uterine horn, effectively restoring the balance between the injured and normal horns. This improvement was paralleled by enhanced embryo development and normalized uterine morphology, suggesting that exosome therapy may offer dual benefits during early disease stages by limiting fibrosis progression while enhancing uterine receptivity and function. Clinically, intrauterine adhesions most commonly develop following surgical curettage, hysteroscopic interventions, or infection‐associated endometrial injury. In such contexts, excessive fibroblast activation and aberrant matrix deposition occur during the early post‐injury repair phase. Therefore, an intervention applied during this early remodelling window to limit pathological fibrotic progression may represent a clinically feasible strategy. Although the present study models early intervention rather than treatment of established adhesions, the dosing paradigm may conceptually align with perioperative or early post‐procedural therapy in patients at high risk of adhesion formation. Future studies will be required to determine whether CAR‐EVs can also reverse mature, matrix‐dense fibrotic lesions.

Despite these promising findings, several challenges remain for the translation of exosome‐based therapies into clinical practice. One of the primary concerns is the standardization of exosome isolation and purification methods, which are critical for ensuring reproducibility and scalability. Furthermore, the complex molecular composition of exosomes poses challenges for elucidating their mechanisms of action and predicting their behaviour in diverse biological environments. In the present study, the dosing regimen was selected based on preliminary experiments demonstrating reproducible antifibrotic effects without overt systemic toxicity, and dosing was normalized to total EV particle number. While exosome therapy effectively reduced fibrotic markers and improved uterine function in this model, systematic dose–response analysis, timing optimization, and long‐term safety evaluation will be required in larger preclinical and clinical studies.

In conclusion, our study highlights both the potential and the challenges of employing immunotherapeutic strategies in the treatment of IUA. CAR‐T‐cell therapy targeting FAP‐positive cells represents a proof‐of‐concept approach that directly addresses the underlying fibrotic process, yet its clinical application is limited by severe CRS‐related toxicity. The concurrent use of IL‐6 blockade, while effective in reducing toxicity, may compromise the therapeutic efficacy of CAR T cells. As an alternative, exosome‐based therapy offers a promising cell‐free approach that mitigates the risks associated with live cell infusions while retaining the capacity to limit fibrosis progression and improve uterine function during early stages following endometrial injury. Future research should focus on optimizing these therapeutic strategies, with an emphasis on understanding the complex interplay between immune activation, inflammation, and tissue repair in IUA. Addressing these challenges will be critical for developing safe and effective treatments that can improve reproductive outcomes and quality of life for patients suffering from intrauterine adhesions.

## 4 Methods

### 4.1 Cell Lines

The NIH/3T3 and 293T cell lines were purchased from the American Type Culture Collection (ATCC). The identities of the cell lines were verified by short tandem repeat analysis. B16.F10 cells were purchased from ATCC. The cells were maintained in DMEM supplemented with 10% foetal bovine serum. Cell culture media and supplements were obtained from Life Technologies (Thermo Fisher Scientific, China).

### 4.2 Vector Construction

As shown in Figure 1b, the CAR design contained the human CD8α signal peptide followed by the scFv linked in frame to the hinge domain of the CD8α molecule, the transmembrane region of the human CD8 molecule, and the intracellular signalling domains of the CD137 and CD3ζ molecules. Unpaired cysteine 164 within the CD8α hinge region was replaced with a serine to increase CAR expression, as reported previously.

### 4.3 Antibody Library

The protocol for antibody library construction has been described in our previous reports. Briefly, peripheral blood mononuclear cells were isolated from human blood. All specimens were collected under an approved protocol by the Second Military Medical University Review Board, and written informed consent was obtained from each donor. Total RNA was extracted, and nested PCR was used to clone genes with single‐domain antibodies consisting of the heavy chain and light chain variable domains. The final PCR products were carried by phagemid vector pCANTAB5E (GE Healthcare), which was introduced into freshly prepared, electrocompetent *Escherichia coli* TG1 cells. The cells were selected on lysogeny broth agar plates supplemented with ampicillin and glucose cultured overnight at 37°C. After being scraped from the plates, the clones were stored at −80°C in lysogeny broth supplemented with 20% glycerol.

### 4.4 In Vitro T‐Cell Transduction and Culture

Murine CD8+ and CD4+ T cells were enriched from the spleen and peripheral lymph nodes of congenic BALB/c mice using untouched negative isolation kits (Stem Cell Technologies) and stimulated with plate‐bound anti‐CD3 and anti‐CD28 antibodies (clones 145‐2C11 and 37.51, respectively) at 1 mg ml^−1^ each for 24 h at 37°C in a 5% CO_2_ incubator in complete RPMI medium supplemented with 50 U ml^−1^ recombinant murine IL‐2 (Peprotech). Murine T cells were collected from anti‐CD3/28‐coated plates and resuspended at 1 × 10^6^ cells per ml in complete RPMI supplemented with 50 U ml^−1^ IL‐2 and anti‐CD3/28 mouse T‐activator Dynabeads (Thermo Fisher) at a bead‐to‐cell ratio of 1:1. The viral supernatant was aspirated from RetroNectin‐coated plates; the plates were then rinsed with PBS, and 1 mL (1 × 10^6^) of T cells were added to each virus‐coated well. The cells were transduced with lentiviral vectors encoding the indicated CAR, iCASP9‐IL‐15 or short hairpin RNA (shRNA) hairpin sequences targeting Tet2 or a scrambled control co‐expressing blue fluorescent protein (BFP) (GeneChem). The plates were then centrifuged at 800 × g for 30 min at 32°C and returned to 37°C in 5% CO_2_ incubators. A second transduction was performed as described the next day. T cells were subsequently collected, counted and resuspended in complete RPMI medium supplemented with 20 ng ml^−1^ IL‐15 every 1–2 day. At 4 days after transduction, the magnetic beads were removed, and T‐cell transduction was measured by flow cytometry staining with the following markers: untransduced T cells, myc−; CAR T cells, myc+GFP+; and CAR T shTet2 cells, myc+GFP+BFP+. The cells were sorted by FACS for enrichment and then normalized by diluting the cultures with untransduced T cells. The knockdown efficiency in T cells following shRNA transduction was determined by real‐time quantitative PCR with TaqMan gene expression assays (Applied Biosystems) for Tet2 (assay Mm00524395_m1), and Gapdh (assay Mm99999915_g1) served as a loading and normalization control.

### 4.5 In Vivo Study

The in vivo experiments were approved by the Institutional Animal Care and Use Committee (IACUC) of Second Military Medical University, and the mice were housed in a specific‐pathogen‐free barrier facility. BALB/c female mice were obtained from Shanghai Model Organisms Center, Inc. FAP^–/–^ (#024288) mice (Niedermeyer et al. 2001) were obtained from the Jackson Laboratory and were maintained by crossing with C57BL/6J wild‐type mice. Eight‐week‐old mice were used for the experimentally induced mouse model of AS, as previously described with minor modifications (Alawadhi et al. 2014). A vertical incision was made in the abdominal wall, and the uterus was exposed. A small incision was made in each uterine horn at the uterotubal junction, and one horn was traumatized in a standardized fashion using a 27‐gauge needle inserted through the lumen; the needle was rotated and withdrawn 10 times. The mice were injected i.p. with 200 mg kg^−1^ cyclophosphamide to deplete the host lymphocyte compartments, and 5–6 h later, different CAR T cells were injected intravenously at a dose of 1 × 10^7^ cells. After cell transplantation in the mouse model of AS, the therapeutic effects were assessed on Day 14. For EV treatment experiments, mice received repeated i.V. injections of CAR‐derived EVs during the early phase following uterine injury (within 6 h after uterine injury induction). EVs were administered every 2 days for a total of 6 doses. Each dose was normalized to total EV particle number (6×10^10^ or 1.2×10^11^ particles per mouse, NTA), corresponding to 75 µg or 150 µg exosomal protein (Bradford; Table S3). The timing and frequency were chosen to assess the capacity of EVs to attenuate fibrotic progression during the injury‐repair window rather than to model treatment of established fibrosis.

### 4.6 Isolation and Purification of Exosomes

Exosomes were purified from cell culture supernatants. Briefly, culture supernatants were centrifuged at 300 ×g for 5 min and then at 1200×g for 20 min and 10,000×g for 30 min to eliminate cells and debris. The supernatants were ultracentrifuged at 100,000 ×g for 60 min at 4°C to pellet the exosomes. The exosome pellets were washed twice in a large volume of PBS and recovered by centrifugation at 100,000 ×g for 1 h. Exosomal protein was measured by the Bradford assay with the Bio‐Rad Protein Assay Reagent (Bio‐Rad, Hemel Hempstead, UK) and stored at −80°C. For further purification of CAR exosomes, those isolated from CAR‐T‐cell culture supernatants were resuspended in PBS. The supernatants were then mixed with FAP—or HER2‐coated Dynabeads (Dynal Biotech, Oslo, Norway). The mixture was incubated overnight at 4°C on a rotating plate, and the beads were collected and washed twice with PBS on a magnetic rack to eliminate unbound or excess exosomes. CAR exosomes were eluted with 0.1 M sodium citrate/citric acid and were immediately equilibrated at the desired pH. In addition to protein quantification by Bradford assay, EV preparations were routinely characterized by size distribution and particle concentration using NTA to ensure batch‐to‐batch consistency. Protein concentration was reported as a complementary metric and used for practical handling, whereas particle number was used to estimate administered EV doses in vivo.

### 4.7 Quantification and Dosing of CAR‐T‐Derived Extracellular Vesicles

EV yield was quantified using nanoparticle tracking analysis (NTA). Briefly, EV preparations isolated from CAR‐T cell culture supernatants were diluted in sterile PBS and analysed using a NanoSight NS300 system (Malvern Panalytical) equipped with a 488‐nm laser. For each sample, three independent recordings of 60 s were acquired, and particle concentrations were calculated using NTA software. Based on NTA measurements, the average EV yield per millilitre of CAR‐T cell culture supernatant was estimated, allowing calculation of the approximate number of EV particles administered per injection. Unless otherwise specified, EV dosing in all in vivo experiments was normalized by total EV particle number, with corresponding protein amounts reported in parallel. We note that CAR‐positive EVs likely represent a subset of the total EV population derived from CAR‐T cells; therefore, dosing reflects total EV preparations rather than exclusively CAR‐expressing vesicles. This limitation is explicitly acknowledged in the manuscript.

### 4.8 Quantitative PCR (qPCR)

Total RNA was isolated using the RNeasy Mini Kit (Qiagen) according to the manufacturer's specifications. Real‐time quantitative PCR was performed on an ABI PRISM 7900HT and analysed using SDSv2.3 (Applied Biosystems). qPCR was performed with the following commercially available TaqMan probes: Col1a1 (Mm00801666_g1), Tgfb1 (Mm01178820_m1), Timp1 (Mm01341361_m1), and Gapdh (Mm03302249_g1).

### 4.9 Cytokine Measurements

Serum/plasma cytokines were measured using cytometric bead arrays (BD) or ELISA kits for mouse SAA3 (Millipore) according to the manufacturer's instructions.

### 4.10 Histological Staining

To investigate the endometrial structure, frozen uterine sections (12 mm) were fixed in 4% paraformaldehyde and stained with haematoxylin and eosin. The collagen areas were stained with Masson's trichrome to evaluate fibrosis. Under Masson's trichrome staining, the collagen fibres appeared blue, and the background appeared red. The slides were scanned using an image scanner (Scan‐Scope AT, Aperio).

### 4.11 High‐Throughput Sequencing of CAR Libraries

V‐gene repertoires isolated from CAR+ cells were sequenced on the Illumina MiSeq platform (GENEWIZ). Processing of the sequencing data was performed as previously described (Asensio et al. 2019). Briefly, the CDR3 amino acid sequences from each scFv sequence were concatenated into a single contiguous amino acid sequence. The sequences were further checked manually to define ‘clones’ conservatively. The total number of amino acid differences in all pairwise alignments between each concatenated sequence in each dataset was further computed. A heatmap was generated to illustrate the prevalence of highly abundant CDRH3s from each sample of the different mouse models. Only CDRH3 sequences with statistically significant frequencies in the top 10 of the distribution were represented.

### 4.12 Immunoprecipitation and Mass Spectrometry

Tissues were lysed in lysis buffer (20 mM Tris‐HCl, pH 7.4; 0.3 M NaCl; and 1% Nonidet P‐40) supplemented with complete protease inhibitor cocktail (Roche). The scFv‐Fc was immobilized onto protein A beads (Thermo Fisher Scientific) and crosslinked with dimethyl pimelimidate dihydrochloride (DMP) (final concentration of 20 mM) (Sigma) at RT for 30 min. The reaction was stopped by incubation in ethanolamine (0.2 M, pH 8.0) at RT for 2 h. The beads were washed with PBS, incubated with cell lysates at 4°C overnight, washed with 0.1% PBS/Tween 20 containing 500 nM NaCl, and then washed (> 3 times) with PBS. The beads with antigens captured were loaded onto SDS‐PAGE gels (4‒20% gradient polyacrylamide) (Thermo Fisher Scientific); one gel was then used for Gelcode staining (Thermo Fisher Scientific), and the other was used for Western blot analysis to locate the membrane protein band by adding streptavidin‐conjugated horseradish peroxidase (HRP) (Jackson ImmunoResearch Laboratories) and ECL substrate (Thermo Fisher Scientific). Bands on the Gelcode‐stained gel corresponding to positions on the Western blot were excised, digested in the gel with trypsin and analysed by tandem mass spectrometry (MS/MS, Mass Spectrometry Facility, University of Minnesota). Spectra were searched using Protein Prospector against the SwissProt database (Ha et al. 2014).

### 4.13 Flow Cytometry

Cell surface staining was performed for 30 min at 4°C, and the samples were analysed using a FACSCalibur flow cytometer (BD Biosciences) and CellQuest software (BD Biosciences). Cellular staining was performed for 60 min on ice after a fixation/permeabilization kit (eBioscience) was used. A minimum of 1 × 10^4^ samples were examined.

### 4.14 Cytotoxicity Assays

The cytotoxicity of the CAR‐expressing T cells or exosomes was tested via a standard 4‐h ^51^Cr release assay (Johnson et al. 2015). The target cells were labelled with 51Cr for 1 h at 37°C. Radioactive ^51^Cr (50 µCi) was used to label 1 × 10^6^ target cells. One hundred microlitres of labelled target cells (*n* = 5000) were plated in each well of a 96‐well plate. Effector cells were added at a volume of 100 µL at different E:T ratios. Exosomes were added at different concentrations. CAR T cells or exosomes and targets were incubated together for 4 h at 37°C. The supernatant (30 µL) from each well was collected and transferred to the filter of a LumaPlate. The filter was allowed to dry overnight. The radioactivity released into the culture medium was measured using a β‐emission‐reading liquid scintillation counter. The percentage of specific lysis was calculated as follows: (sample counts—spontaneous counts)/(maximum counts—spontaneous counts) × 100.

### 4.15 Statistical Analysis

Unless otherwise specified, Student's *t*‐test was used to evaluate the significance of differences between 2 groups, and ANOVA was used to evaluate differences among 3 or more groups. Differences between samples were considered statistically significant when *P* < 0.05.

## Author Contributions

**Wenyan Fu**: conceptualization, methodology, software, data curation, investigation, formal analysis, supervision, funding acquisition, validation, visualization, project administration, resources, writing – original draft, writing – review and editing. **Kewen Qian**: methodology, data curation, validation, formal analysis. **Hongru Ai**: methodology, data curation, investigation, validation, formal analysis. **Yitan Zou**: methodology, data curation, investigation, validation, formal analysis. **Yaping Zhou**: methodology, data curation, investigation, validation, formal analysis. **Ruixue Mao**: methodology, data curation. **Jian Zhao**: methodology, data curation, supervision. **Changhai Lei**: methodology, data curation, investigation, supervision, funding acquisition. **Shi Hu**: writing – review and editing, writing – original draft, resources, project administration, visualization, funding acquisition, supervision.

## Conflicts of Interest

The authors declare no conflicts of interest.

## Supporting information




**Supporting material**: jev270284‐sup‐0001‐SuppMat.docx

## Data Availability

The authors declare that the data supporting the findings of this study are available within the paper and its Supplementary Information files or from the corresponding author on reasonable request.
