# Supplementary material for: Development of CAR Exosomes Targeting FAP for the Treatment of Intrauterine Adhesion
Source: J Extracell Vesicles. 2026 Apr 30;15(5):e70284. doi: 10.1002/jev2.70284 (PMC13132341; doi:10.1002/jev2.70284)
Supplement: Supplementary file 1 — Supporting material: jev270284‐sup‐0001‐SuppMat.docx [file JEV2-15-e70284-s001.docx]

# Supplementary Figures


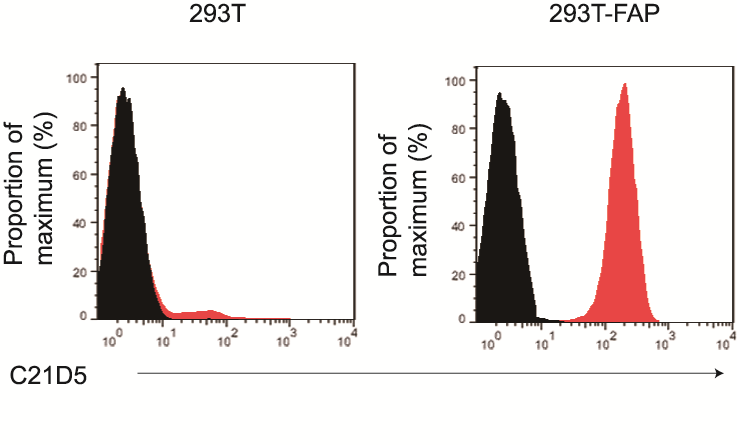


**Figure S1. Antibody bound to FAP.** The expression of FAP on differnet 293T cells was measured by staining with C21D5 antibody, followed by flow cytometry analysis. The histograms shown in black correspond to the isotype controls, whereas the red histograms indicate positive fluorescence. Data are representative of at least three independent experiments.


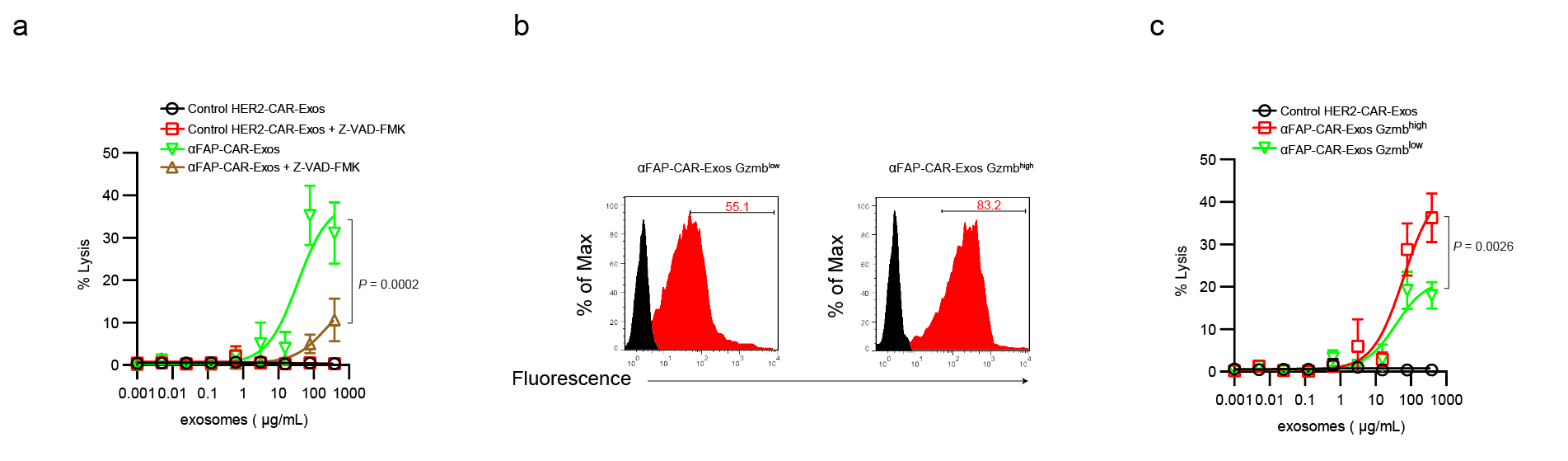


**Figure S2. Granzyme B abundance is associated with enhanced CAR-EV cytotoxicity.** (a) Target cell lysis induced by αFAP-CAR exosomes in the presence or absence of Z-VAD-FMK, measured by Cr^51^ release assay. (b) Flow cytometry analysis showing differential granzyme B levels in GzmB^low^ and GzmB^high^ αFAP-CAR exosome preparations. (c) Dose-dependent target cell lysis induced b αFAP-CAR exosomes compared with control HER2-CAR exosomes. Data are presented as mean ± s.e.m.; *P* values are indicated.

**
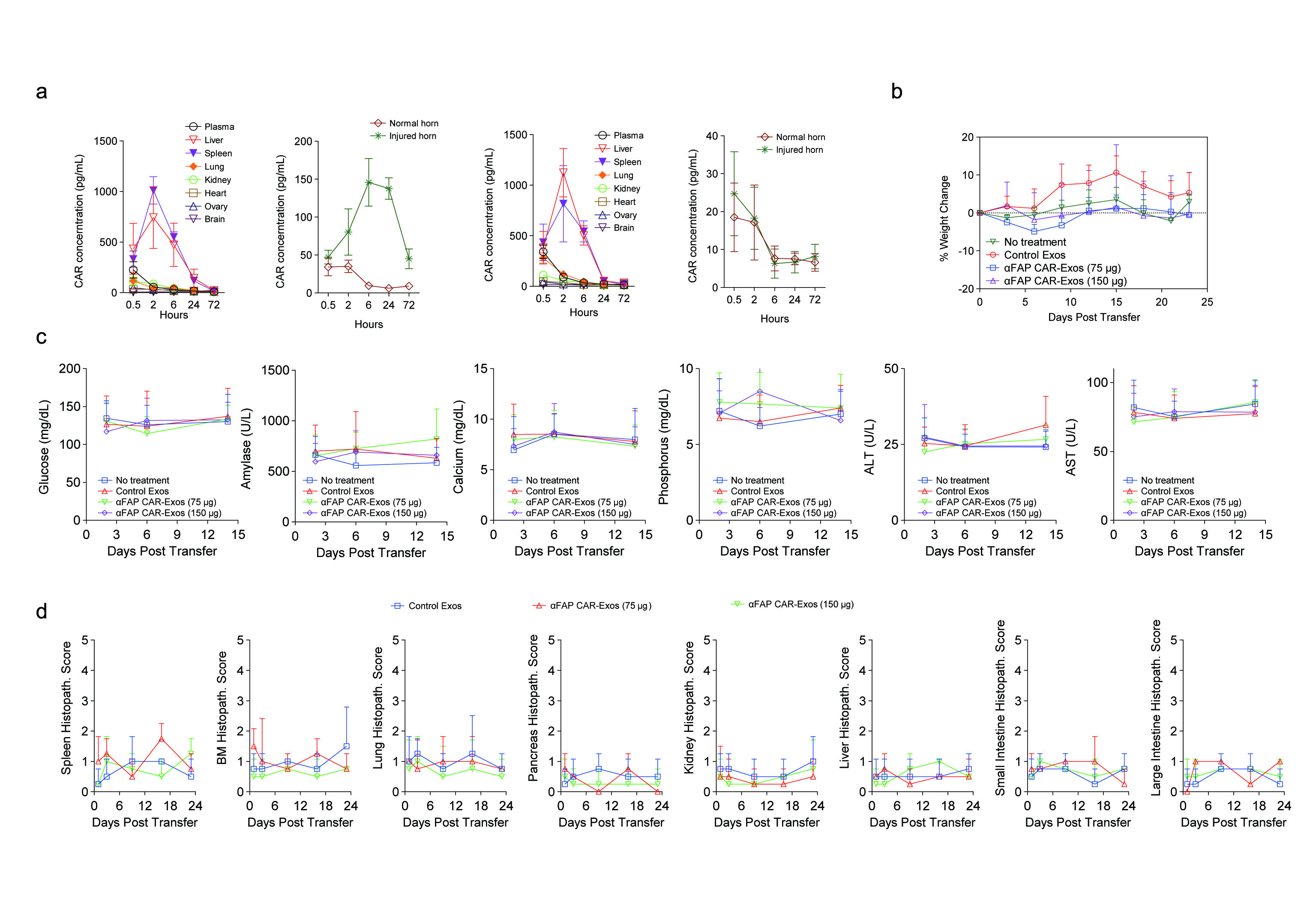
**

**Figure S3. Mechanistic and safety evaluation of** α**FAP-CAR-derived extracellular vesicles. (**a) In vivo biodistribution of CAR-EVs following intravenous administration. CAR-associated signals were quantified in major organs at indicated time points post-injection, demonstrating transient tissue distribution and progressive clearance. (b) Body weight monitoring of mice receiving repeated CAR-EV injections. (c) Serum chemistry analysis. ALT = alanine aminotransferase. AST = aspartate aminotransferase. (d) Histopathology scoring of different treatment. See supplementary table 4 for the histopathology scoring system.


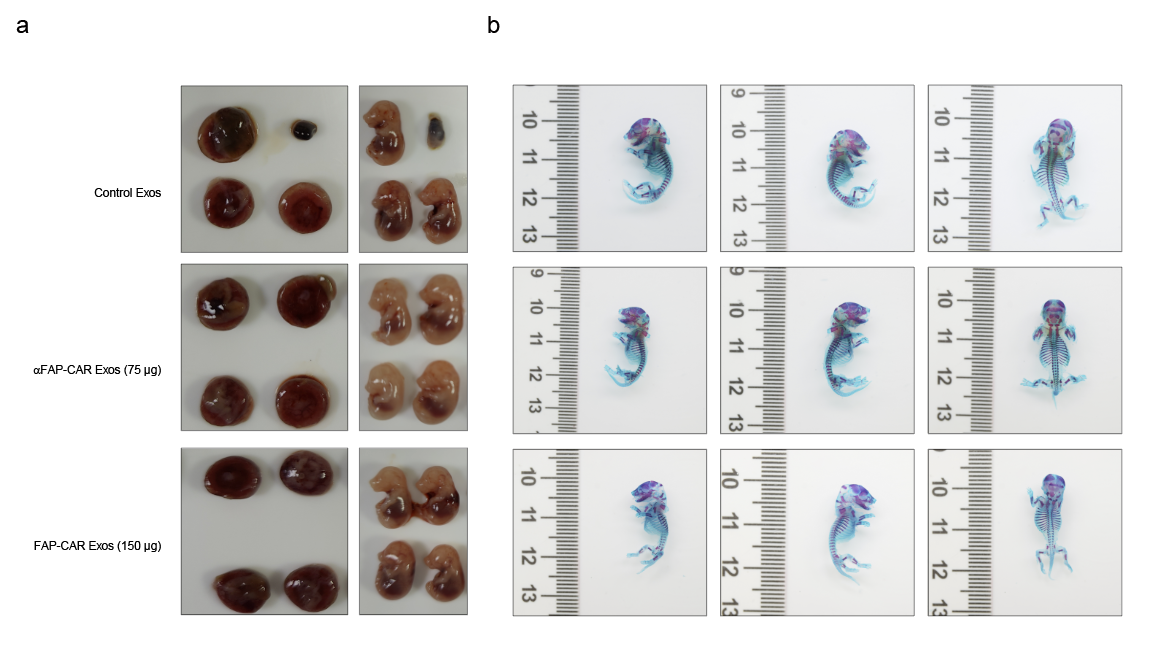


**Figure S4. EVs improve Embryo Implantation and do not affect Subsequent Developments. a**. Representative images of embryos and their placentas retrieved from the injured uterine horn of ISs. **b.** Alcian blue and Alizarin red staining of embryos.

# Supplementary Tables

### Table S1. Selected analytical and affinity data of antibodies.

| Parameter | Unit | C21D5 | Clone 73.3 |
| --- | --- | --- | --- |
| HMW formation after storage* | % SEC area | < 0.1 | < 0.1 |
| LMW formation after storage* | % SEC area | < 0.1 | < 0.1 |
| Affinity/kinetics of S binding** (SPR) | Ka (1/Ms) | 9.44 × 10^4^ | 6.08 × 10^5^ |
|  | Kd (1/s) | 4.95 × 10^-4^ | 1.43 × 10^-3^ |
|  | KD (nM) | 5.24 | 3.74 |

*Quiescent storage for 4 week, 40 °C, 1 mg/mL

**Affinity KD analyzed using a BIAcore T100. The data were globally analyzed using a simultaneous fit for both dissociation (kd) and association (ka). The value for KD was calculated as kd/ka (nanomolar, nM).

### Table S2. Participant characteristics.

| **Characteristics** | Modified AFS Stage | | | | p Value |
| --- | --- | --- | --- | --- | --- |
|  | 0 | I (mild) | II(moderate) | III (severe) |  |
| **Characteristics of Patients** |  |  |  |  |  |
| Age (years) | 37.8 ± 4.1 | 39.69 ± 6.20 | 39.81 ± 3.54 | 37.93 ± 3.99 | 0.523 |
| Body mass index (Kg/m2) | 22.57 ± 2.34 | 23.40 ± 2.62 | 22.57 ± 2.23 | 38.91 ± 4.50 | 0.574 |
| ***Previous Uterine Surgery** |  |  |  |  | 0.003 |
| No | 4 (40%) | 3 (23.1%) | 1 (6.3%) | 0 (0.%) |  |
| Yes | 6 (60%) | 10 (76.9%) | 15 (93.8%) | 14 (100) |  |
| **Abortion History** |  |  |  |  | 0.743 |
| No | 4 (40%) | 3 (23.1%) | 5 (31.3%) | 3 (21.4%) |  |
| Yes | 6 (60%) | 10 (76.9%) | 11 (68.8%) | 11 (78.6 %) |  |
| **Gravidity** | 1.10 ± 0.738 | 1.77 ± 1.17 | 2.06 ± 0.92 | 2.00 ± 0.68 | 0.057 |
| **Gravidity, times** |  |  |  |  | 0.393 |
| 0 | 2 (20%) | 2 (15.4%) | 0 (0%) | 0 (0%) |  |
| 1 | 5 (50%) | 3 (23.1%) | 5 (31.3%) | 3 (21.4%) |  |
| 2 | 3 (30%) | 5 (38.5%) | 6 (37.5%) | 8 (57.1%) |  |
| 3 | 0 (0%) | 2 (15.4%) | 4 (25%) | 3 (21.4%) |  |
| 4 | 0 (0%) | 1 (7.7%) | 1 (6.3%) | 0 (0%) |  |
| **Parity** | 0.50 ± 0.53 | 0.69 ± 0.48 | 0.81 ± 0.54 | 0.64 ± 0.50 | 0.050 |
| **Parity, times** |  |  |  |  | 0.688 |
| 0 | 5 (50%) | 4 (30.8%) | 4 (25.0%) | 5 (35.7%) |  |
| 1 | 5 (50%) | 9 (69.2%) | 11 (68.8%) | 9 (64.3%) |  |
| 2 | 0 (0%) | 0 (0%) | 1 (6.3%) | 0 (0%) |  |

### Table S3. Quantification of CAR-T-derived extracellular vesicles

| Parameter | Value (approximate) | Method |
| --- | --- | --- |
| EV isolation source | CAR-T cell culture supernatant | – |
| EV isolation method | Differential ultracentrifugation | – |
| Mean particle diameter | ~80–100 nm | NTA / TEM |
| EV yield | ~10⁹ particles / mL culture | NTA |
| EV protein content | ~1.25 μg / 10^9^ particles (range 0.5–3 μg/10⁹) | Bradford assay + NTA normalization |
| EV dose per injection | 6×10¹⁰ or 1.2×10¹¹ particles per mouse (corresponding to 75 or 150 μg protein) | Calculated from NTA-based particle quantification |
| Dosing normalization | Normalized by total EV particle number (NTA-based) | – |
| CAR⁺ EV proportion | Not directly quantified; functional dosing based on total EV particle count | – |

### Table 4. Histopathology scoring system of tissues.

|  | Histology Score | | | | |
| --- | --- | --- | --- | --- | --- |
|  | 0 | 1 | 2 | 3 | 4 |
| Spleen | Within  normal  limits | Minimal RP/WP*  atrophy with focal  regeneration | Moderate RP/WP*  atrophy | Severe RP/WP*  atrophy and  hyperplasia | Severe RP/WP*  atrophy and  tissue necrosis |
| BM | Within  normal  limits | Minimal marrow  atrophy with  myeloid  regeneration | Moderate marrow  atrophy with myeloid  regeneration | Moderate marrow  atrophy with  myelofibrosis and  focal myeloid  regeneration | Severe marrow  atrophy with  myelofibrosis and  no myeloid or  erythroid  regeneration |
| Liver | Within  normal  limits | Minimal multifocal  cholangiohepatitis | (not observed) | (not observed) | (not observed) |
| Lung | Within  normal  limits | Minimal multifocal  perivasculitis | (not observed) | (not observed) | (not observed) |
| Kidney | Within  normal  limits | Mesangial  glomerulopathy | (not observed) | (not observed) | (not observed) |
| Pancreas | Within  normal  limits | (not observed) | (not observed) | (not observed) | (not observed) |
| Small  Intestine | Within  normal  limits | Moderate  lymphoid atrophy | Severe lymphoid  atrophy and villous  atrophy/fibrosis | (not observed) | (not observed) |
| Large  Intestine | Within  normal  limits | Moderate  lymphoid atrophy | Severe lymphoid  atrophy with focal  ulcers | (not observed) | (not observed) |

*RP = red pulp; WP = white pulp
